# Durable contraception in the female domestic cat using viral-vectored delivery of a feline anti-Müllerian hormone transgene

**DOI:** 10.1038/s41467-023-38721-0

**Published:** 2023-06-06

**Authors:** Lindsey M. Vansandt, Marie-Charlotte Meinsohn, Philippe Godin, Nicholas Nagykery, Natalie Sicher, Motohiro Kano, Aki Kashiwagi, Maeva Chauvin, Hatice D. Saatcioglu, Julie L. Barnes, Amy G. Miller, Amy K. Thompson, Helen L. Bateman, Elizabeth M. Donelan, Raquel González, Jackie Newsom, Guangping Gao, Patricia K. Donahoe, Dan Wang, William F. Swanson, David Pépin

**Affiliations:** 1 Center for Conservation and Research of Endangered Wildlife (CREW), Cincinnati Zoo & Botanical Garden, Cincinnati, OH USA; 2 Pediatric Surgical Research Laboratories, Massachusetts General Hospital, Department of Surgery, Harvard Medical School, Boston, MA USA; 3 Horae Gene Therapy Center, University of Massachusetts Chan Medical School, Worcester, MA USA

**Keywords:** Gene therapy, Genetic engineering, Reproductive biology, Target validation

## Abstract

Eighty percent of the estimated 600 million domestic cats in the world are free-roaming. These cats typically experience suboptimal welfare and inflict high levels of predation on wildlife. Additionally, euthanasia of healthy animals in overpopulated shelters raises ethical considerations. While surgical sterilization is the mainstay of pet population control, there is a need for efficient, safe, and cost-effective permanent contraception alternatives. Herein, we report evidence that a single intramuscular treatment with an adeno-associated viral vector delivering an anti-Müllerian hormone transgene produces long-term contraception in the domestic cat. Treated females are followed for over two years, during which transgene expression, anti-transgene antibodies, and reproductive hormones are monitored. Mating behavior and reproductive success are measured during two mating studies. Here we show that ectopic expression of anti-Müllerian hormone does not impair sex steroids nor estrous cycling, but prevents breeding-induced ovulation, resulting in safe and durable contraception in the female domestic cat.

## Abstract (web summary)

This study demonstrates the safety and long-term efficacy of a single-dose, injectable contraceptive in female domestic cats. Treated females remained contracepted for over two years, and did not ovulate or produce kittens when paired with males.

## Introduction

Anti-Müllerian hormone (AMH) (or Müllerian inhibiting substance, MIS) is a member of the transforming growth factor-beta (TGFβ) superfamily of ligands that is crucial for sex determination in fetal development^1^. In females, both AMH and its type II receptor (AMHR2) are highly expressed in granulosa cells of ovarian follicles^2–4^, including those of the feline ovary^5–7^. We have recently demonstrated that supraphysiological levels of AMH, resulting from gene therapy with adeno-associated virus (AAV) vector, can suppress folliculogenesis and induce permanent contraception in adult female mice^8^. The mechanism of contraception is unlike other hormonal contraceptives, acting primarily on the early gonadotropin-independent steps of follicle development, inhibiting both primordial follicle activation and the maturation of pre-antral follicles^9^.

For wildlife population control, several contraceptive strategies have been extensively evaluated including vaccines^10^, and other non-surgical methods^11,12^ such as sex steroid analogs, Gonadotropin-Releasing Hormone (GnRH) agonists and antagonists, and physical barrier devices. These approaches have not to date been shown to represent effective long-term contraception capable of replacing surgical sterilization methods^13–15^, and none are currently approved by the Food and Drug Administration (FDA) or the European Medicines Agency (EMA) for contraception or sterilization of female or male cats. However, progress in the development and refinement of AAV-based gene therapy for human use^16,17^ has expanded opportunities to apply this technology to animal contraception^18,19^.

In this study we use AAV9 to deliver a domestic cat (*Felis silvestris catus)* AMH transgene (termed fcMISv2) in adult female domestic cats. We show that this vectored contraceptive prevents breeding-induced ovulation, results in complete infertility, and may constitute a safe and durable strategy to control reproduction in the domestic cat.

## Results

### Optimization of feline AMH transgene and validation of AAV9 vector

**Fig. 1:**
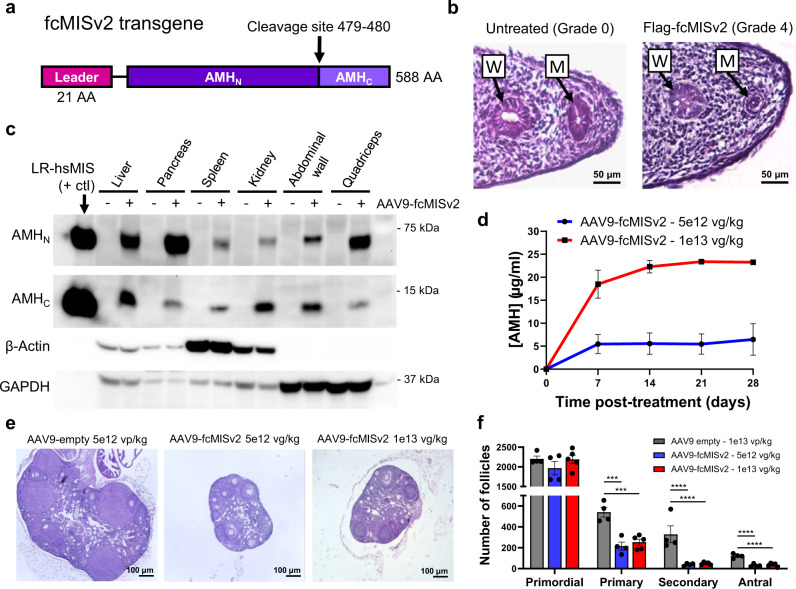
Cloning of a feline-specific AMH transgene and validation of AAV9-fcMISv2 in mice. **a** Design of a codon optimized feline AMH transgene (fcMISv2). AMH_N_ is the N-terminal prodomain, and AMH_C_ is the C-terminal mature domain of the AMH protein. **b** Fetal rat urogenital ridge sections stained with H&E of untreated female ridge (negative control), and female ridge treated with 5 µg/ml of purified feline AMH protein (in vitro cleaved FLAG-fcMISv2). Urogenital ridges representative of three independent experiments are shown. Activity of AMH is scored from grade 0–5 representing the degree of Müllerian duct regression. W = Wolffian duct, M = Müllerian duct. **c** Western blot of a panel of tissue lysates from mice 30 days after treatment with 1e13 viral genomes per kilogram (vg/kg) of AAV9-fcMISv2 (+) or with 5e12 viral particles per kilogram (vp/kg) of AAV9-empty vector control (−). Human AMH (LR-hsMIS) acts as a positive control. β-actin and GAPDH were used as loading controls. **d** Serum concentration of AMH measured by ELISA in mice following treatment with 5e12 vg/kg (blue, circles) or 1e13 vg/kg (red, squares) of AAV9-fcMISv2. *n* = 3 biologically independent animals. Data are presented as mean values ± SEM. **e** Representative middle section of the ovaries of mice four weeks after treatment with 5e12 vg/kg or 1e13 vg/kg of AAV9-fcMISv2, or 5e12 vp/kg of AAV9-empty vector control. **f** Follicle counts of whole ovaries at the same timepoint. Mice were treated with 5e12 vg/kg (blue) or 1e13 vg/kg (red) of AAV9-fcMISv2, or 5e12 vp/kg of AAV9-empty vector control (grey) (*n* = 4, 5, and 4 per group, respectively). One-way ANOVA (Dunnett’s post hoc test): ***P = 0.0003 (5e12 vg/kg*)* and 0.0005 (1e13 vg/kg) for primary follicles. ****P < 0.0001 for secondary and antral follicles. Source data are provided as a Source Data file.

To adapt vectored contraception with AMH to the domestic cat, we designed a first-generation feline transgene, termed fcMISv1, which was constructed using the (at the time) incomplete AMH sequence of the cat genome version 8.0^20^. The gaps in the AMH sequence were filled using carnivora consensus sequence. Subsequently a second-generation *Felis catus* AMH transgene, termed fcMISv2, was produced based on the domestic cat genome version 9.0^21^ that had a complete AMH sequence (Fig. 1a). This second-generation transgene corrected 31 amino acid differences that were introduced during the construction of fcMISv1 (Supplementary Fig. 1A). Both the fcMISv1 and fcMISv2 transgenes were optimized to use favored feline codons and to reduce their GC content using the GenSmart™ algorithm^22^, and then cloned into mammalian expression vectors (pcDNA3.1), with an introduced N-terminal FLAG-tag on the mature domain (termed AMH_C_) for purification purposes as previously described^23,24^. Both the purified and enzymatically cleaved FLAG-fcMISv1 and FLAG-fcMISv2 (Fig. 1b), were able to induce regression of the Müllerian duct in the fetal rat urogenital ridge bioassay, an ex vivo gold standard of AMH activity^25^, to a grade of 4 (out of 5) at 5 µg/ml. FLAG tag-free versions of the fcMISv1 and fcMISv2 transgenes were cloned into AAV packaging plasmids that included a strong CMV enhancer, a ubiquitous chicken β-actin promoter, a synthetic intron, and a terminating 3’UTR with a rabbit β-globin polyadenylation signal for efficient expression in vivo^26^.

Given the favorable tropism of the AAV9 serotype to muscle tissues^27^ and the relatively low abundance of pre-existing anti-AAV9 antibodies in cats^28,29^, this vector was chosen to deliver the fcMISv1, and later fcMISv2, into this species. We evaluated the AAV9-fcMISv2 vector by intraperitoneal (IP) injection in nude mice to avoid potential transgene immunogenicity. One month following AAV9 treatment we found AMH protein expression in several tissues, including quadriceps and abdominal wall muscles, and peritoneal organs such as kidney, spleen, pancreas, and liver (Fig. 1c), where it was efficiently cleaved by endogenous proteases, resulting in supraphysiological levels in the blood (Fig. 1d). At this 1-month timepoint, the ovaries of mice treated with either 5e12 vector genome per kilogram (vg/kg) or 1e13 vg/kg of AAV9-fcMISv2 were smaller than controls receiving 5e12 AAV9-empty vector particles per kilogram (vp/kg) (Fig. 1e). Ovaries of AAV9-fcMISv2-treated mice displayed a significant reduction in primary, secondary, and antral follicles compared to controls (Fig. 1f), as previously reported in similar experiments using the human AMH transgene^8^.

### Administration of a single dose of a first-generation AAV9-fcMISv1 vector in cats results in prolonged AMH expression in muscles, variable anti-transgene antibody response, and histological changes in the reproductive tract

We sought to evaluate the safety and efficacy of delivering AMH using the first-generation AAV9-fcMISv1 vector, in a pilot study of three female domestic cats treated by intramuscular (IM) injection at a dose of 5e12 vg/kg. No signs of pain, heat, redness, or swelling of the injection sites were observed. No significant adverse findings were noted during regular physical exams and blood works. C-reactive protein levels (CRP, a marker of inflammation) increased slightly 7 days post-injection but returned promptly to baseline levels thereafter (Supplementary Fig. 1B). We monitored the levels of AMH by ELISA (detecting both endogenous and recombinant forms) and developed a direct antigen-antibody capture ELISA to measure anti-fcMISv1 antibodies in the serum, during an extended period of 4 years (Supplementary Fig. 1C). Additionally, to look at the effects of supraphysiological levels of AMH on the cat reproductive tract, we spayed the three females 42 months post-treatment (at 9–10 years of age) and examined their ovaries and uteri histologically (Supplementary Fig. 1D). One female (“Antilles”) produced no detectable antibodies against the fcMISv1 protein and retained high serum AMH protein levels (Supplementary Fig. 1C). Her reproductive tract displayed normal uterine endometrium (Supplementary Fig. 1D). In contrast, histological analysis of “Catalina” and “India”, which developed anti-fcMISv1 antibodies and had lower serum AMH levels (Supplementary Fig. 1C), revealed marked cystic endometrial hyperplasia typical of aged intact cats^30^ (Supplementary Fig. 1D). “India”, which had the strongest antibody response and failed to maintain AMH expression, displayed evidence of both cystic endometrial hyperplasia and multiple corpora lutea in the ovary indicative of spontaneous ovulation (as this female was never housed with a male).

Following euthanasia of “India” due to a traumatic injury unrelated to the study, transgene expression was assessed in its skeletal muscle and liver tissue, as both tissues are known to be highly transduced by AAV9^27^. In situ RNA hybridization detected high fcAMHv1 transgene expression in skeletal myocytes at 49.5 months post-treatment (Supplementary Fig. 2A). In contrast, fcAMHv1 transgene expression in hepatocytes was minimal (Supplementary Fig. 2B). Taken together, these results suggest that AAV9-fcMISv1 efficiently transduced skeletal muscle cells, and that the resulting supraphysiological levels of AMH in “Antilles” suppressed ovulation and cystic endometrial hyperplasia. However, low levels of AMH in the serum of “Catalina” and “India”, likely due to a variable antibody response against the introduced carnivora consensus sequence in the fcMISv1 transgene (Supplementary Fig. 1A), precluded evaluation of its physiological impacts in these cats.

### Administration of a single dose of AAV9-fcMISv2 results in continuous secretion of supraphysiological AMH, with no adverse effect or immune response

**Fig. 2:**
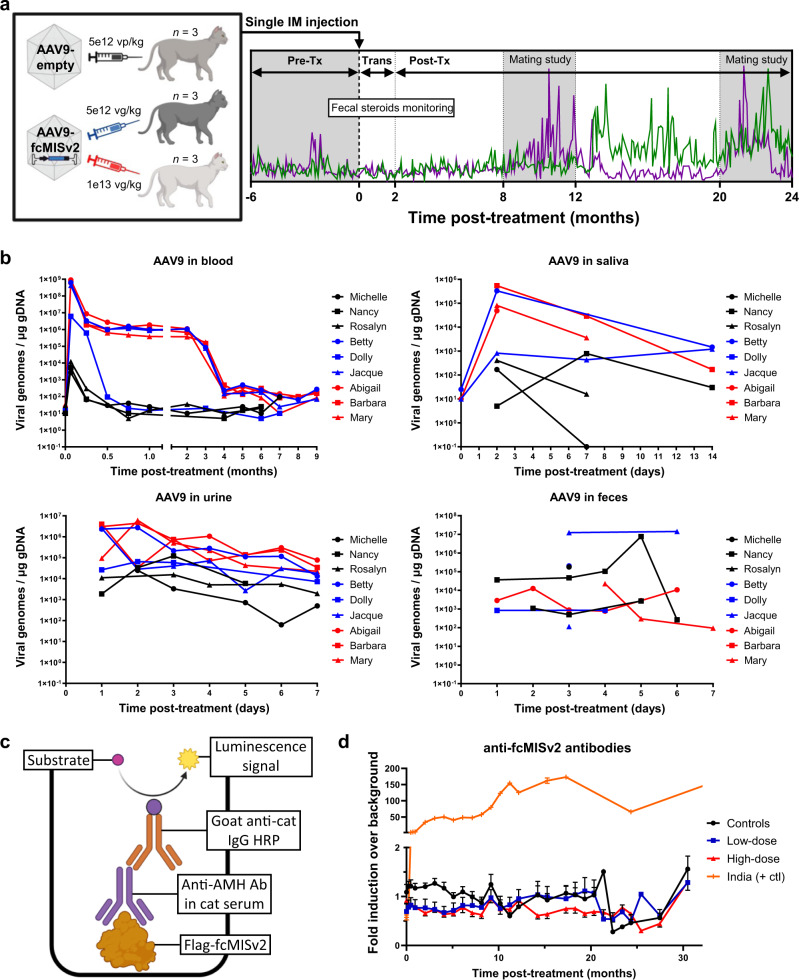
Study timeline and safety of AAV9-fcMISv2 injection in domestic cats. **a** Nine sexually mature female domestic cats were treated intramuscularly with 5e12 (low-dose, blue) or 1e13 (high-dose, red) vg/kg of AAV9-fcMISv2, or 5e12 vp/kg of AAV9-empty vector (control, black). Fertility was assessed during two mating studies (shaded background) concluding at the 1-year and 2-year post-treatment marks. The pre-treatment period (Pre-Tx) spanned the 6 months prior to injection and was followed by a 2-month transition period (Trans), and by a subsequent 22-month post-treatment period (Post-Tx). The green and purple lines are an example of estradiol and progesterone fecal excretion monitoring that spanned the whole study. **b** Viral genome quantification by qPCR in blood, oral swab, urine, and stool samples from domestic cats following injection with low- (blue) or high-dose (red) AAV9-fcMISv2 or AAV9-empty vector control (black). **c** Graphical representation of the designed direct capture protein ELISA assay to measure anti-fcMISv2 IgG in cat serum. **d** Mean serum anti-fcMISv2 antibody titers in female cats injected with low- (blue squares) or high-dose (red triangles) AAV9-fcMISv2 or AAV9-empty vector control (black circles). *n* = 3 biologically independent animals. Data are presented as mean values ± SEM. Serum samples of “India” (orange plus signs), a female that raised an immune response against fcMISv1, is used as a positive control. Left panel in **a** and panel **c** were created with BioRender.com. Source data are provided as a Source Data file.

To evaluate the effect of supraphysiological levels of AMH on feline reproduction we constructed a second-generation vector, AAV9-fcMISv2, using the corrected domestic cat genome^21^ version 9.0 (Fig. 1a). We treated nine cats (*n* = 3 per group) with AAV9-fcMISv2 (5e12 vg/kg, low-dose; 1e13 vg/kg, high-dose) or AAV9-empty vector particles (5e12 vp/kg, control; Fig. 2a).

The vector was delivered intramuscularly in the right caudal thigh muscle. Female cats were assessed daily for general wellbeing during the first two weeks post-injection. Injection sites were also examined daily for 14 days, weekly for the next two weeks, and then monthly thereafter. One cat in the 1e13 vg/kg group developed slight edema at the injection site on Days 3 and 4 that resolved by Day 5. No pain behaviors, increases in temperature, or tissue mass formations were observed. No other injection site reactions were observed. Physical exams and blood work were performed two weeks prior to treatment, at Day 0 (before AAV9-fcMISv2 injection), and then repeated every 3 months through Year 1 of the study, and every 6 months thereafter. No significant adverse findings were observed during physical exams. No evidence of sustained hematologic or biochemical aberrations were observed in blood parameters. More specifically, female cats presented no clinically relevant increases of markers of renal (blood urea nitrogen [BUN], creatinine, symmetric dimethylarginine [SDMA]), liver (alanine aminotransferase [ALT], aspartate aminotransferase [AST]), or skeletal muscle (creatine kinase) damage throughout the study.

Viral shedding in bodily fluids was measured by quantitative PCR. Viral genomes could be readily detected in the blood on Day 2 and remained elevated for 2 months before rapidly decreasing at the 3–4-month period, likely reflecting infected cell turnover (Fig. 2b). Acutely, viral shedding could be detected in the urine on Day 1 and steadily decreased over 7 days, while measurements in oral swabs and stool samples were more variable across individuals and groups (Fig. 2b).

Using purified recombinant FLAG-fcMISv2 protein, we developed a direct antigen-antibody capture ELISA to measure anti-fcMISv2 IgG in domestic cat serum (Fig. 2c). Unlike the pronounced anti-transgene antibody response observed in two of the three cats treated with 5e12 vg/kg of AAV9-fcMISv1 in the first pilot study (Supplementary Fig. 1C), no anti-fcMISv2 antibodies were detected over background in any of the six cats given the optimized AAV9-fcMISv2 vector (Fig. 2d and Supplementary Fig. 3).

**Fig. 3:**
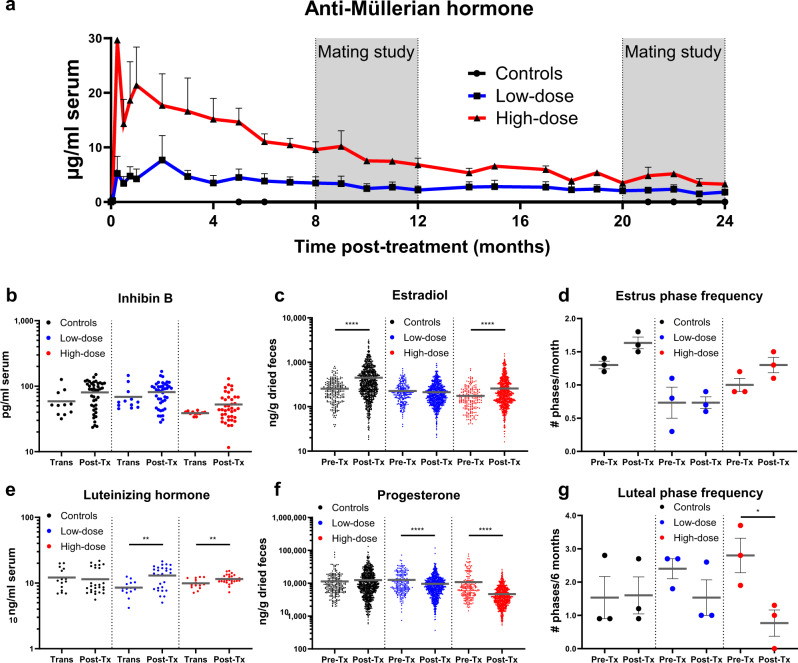
Reproductive hormones following AAV9-fcMISv2 injection in domestic cats. **a** Mean serum concentrations of AMH in cats treated with 5e12 (low-dose, blue line) or 1e13 (high-dose, red line) viral genomes per kilogram (vg/kg) of AAV9-fcMISv2, or 5e12 viral particles per kg of AAV9-empty vector (control, black), were assessed monthly throughout the study. **b** Inhibin B levels in low- (blue) and high-dose (red) groups, and in cats injected with 5e12 AAV9-empty vector particles per kilogram (vp/kg) (controls, black). Samples were compared between the transition period (Trans, 0–2 months post-injection) and the post-treatment period (Post-Tx, 2–24 months post-injection) for each group of cats. **c** Concentrations of estradiol (E2) metabolites in dried fecal samples collected during the pre-treatment (Pre-Tx, up to 6 months prior to the injection) and post-treatment periods were compared for each group of cats. **d** Assessment of estrous phase ( ≥2 consecutive samples with E2 > 1.5 standard deviations over baseline mean) frequency based on fecal steroid profiles in pre- and post-treatment periods. **e** Luteinizing hormone (LH) levels in the blood and **f** progesterone (P4) metabolites in dried fecal samples. **g** Assessment of luteal phase (≥6 consecutive samples with P4 > 1.5 times baseline mean) frequency based on fecal steroid profiles. Horizontal lines in **b**–**g** indicate the mean. Error bars in **a**, **d**, and **g** represent the standard error of the mean. Log-transformed inhibin B, E2, LH and P4 data were compared using an unpaired two-tailed Student’s *t* test for each group of cats: **P = 0.0040 (low-dose) and 0.0076 (high-dose) for luteinizing hormone. ****P < 0.0001 for estradiol and progesterone. Estrous and luteal phase frequencies from pre- and post-treatment periods were compared using a two-way ANOVA (Tukey’s post hoc test): *P = 0.0263 for luteal phase frequency in the high-dose group. *n* = 3 biologically independent animals for each experiment. Source data are provided as a Source Data file.

Circulating AMH levels in the serum were robust but gradually decreased during the first year, eventually reaching a plateau in the second year (Fig. 3a), which remained above our target concentration of 0.25 µg/ml in every cat (Supplementary Fig. 3).

### Supraphysiological AMH induces changes in reproductive hormones and ovarian cycling

To evaluate the effect of supraphysiological AMH on the growing follicle pool, we compared mean serum inhibin B levels in the low-dose, high-dose, and AAV9-empty vector control groups at two time periods. The first time period consists of samples collected during a 2-month “transition” period immediately following AAV9-fcMISv2 treatment (Fig. 2a), which we hypothesized corresponds to the duration needed for the suppressive effects of AMH on the ovary to be fully manifested^13,31^. The second time period covers samples taken from 2 to 24 months post-treatment (Fig. 2a), but excludes periods of mating, pregnancy, and lactation when blood collections were suspended to minimize disruption to cat behavior. Unlike similar experiments in the mouse^13,31^, we did not observe a significant decline of inhibin B in cats following treatment with AAV9-fcMISv2 (Fig. 3b), suggesting pre-antral and early antral follicle hormones may be unaffected by supraphysiological AMH.

Next, we evaluated antral follicle hormones. First, we measured estradiol (E2) metabolites in fecal samples collected thrice weekly throughout the study. We compared the mean E2 level of each group of cats between the pre-treatment (Fig. 2a) and the post-treatment periods. Supraphysiological levels of AMH did not result in a decreased estradiol excretion in female cats injected with AAV9-fcMISv2. Rather, probably due to regular uninterrupted estrous-interestrous cycles, both the control and the high-dose AAV9-fcMISv2 groups presented significantly elevated E2 fecal excretion during the post-treatment period (*P* < 0.0001 for both; Fig. 3c). No increase in E2 was observed in the low-dose group. To better understand the follicular populations and their hormonal activity in cats treated with AAV9-fcMISv2, we assessed several other ovarian hormones in serum samples. Levels of inhibin A and follistatin, both produced by granulosa cells of secondary follicles onward^32^ were similar in the high-dose group of cats between the transition and post-treatment periods (Supplementary Fig. 4A). Follistatin levels were significantly lower in the control group during the post-treatment compared to the transition period (*P* < 0.0001). In the female mammal, testosterone (T) is mainly produced by theca cells of growing follicles. To evaluate if AMH affected theca cell function we measured T in our animals^8,33^; we did not observe a decline in testosterone serum levels following treatment with a low- or high-dose of AAV9-fcMISv2 (Supplementary Fig. 4B).

To evaluate the effect of supraphysiological levels of AMH on estrous cycling, we used fecal E2 levels to infer the timing of estrous phases (Fig. 3d and Supplementary Fig. 5). An estrous phase was defined as two or more consecutive fecal samples with an E2 value greater than 1.5 standard deviations over the baseline mean. No differences were observed in the estrous phase frequency between the pre- and post-treatment periods for any group (Fig. 3d), suggesting cyclic antral follicle secretion of E2 is unaffected by supraphysiological AMH. In order to indirectly estimate the abundance of follicles of more advanced antral stages^34,35^, we measured C-type natriuretic peptide (CNP) and compared the transition and post-treatment periods for each group of cats. CNP levels were elevated in control cats during the post-treatment period (*P* = 0.045) but were unchanged in AAV9-fcMISv2 treated females of both low- and high-dose groups (Supplementary Fig. 4C). These data may reflect a greater abundance of large preovulatory follicles during the post-treatment period in control females that is not observed in cats treated with AAV9-fcMISv2. Together these results indicate that the populations of antral follicles present in the ovaries of cats treated with AAV9-fcMISv2 can maintain hormone production and estrous cycling.

Next, to evaluate the effect of supraphysiological AMH on the hypothalamic-pituitary-gonadal axis and ovulation we measured serum LH in the low-dose, high-dose, and AAV9-empty vector control cohorts. Analysis revealed that the LH levels were elevated in the post-treatment period in comparison to the transition period in the low- and high-dose AAV9-fcMISv2-treated cats (*P* = 0.004 and *P* = 0.008, respectively) but not controls (Fig. 3e). Following ovulation, granulosa and theca cells from the ovulated follicle differentiate into progesterone-producing luteal cells. To evaluate if ovulation occurred in cats treated with AAV9-fcMISv2 we measured progesterone (P4) metabolites levels in fecal samples collected three times a week throughout the study. Mean P4 concentration was significantly reduced in both the low- and high-dose groups following treatment (*P* < 0.0001 for both), whereas the control group did not differ between the pre- and post-treatment periods (Fig. 3f). Together, these results suggest a mild hypergonadotropic hypogonadism phenotype with reduced P4 and elevated LH in AAV9-fcMISv2 treated cats.

In the female domestic cat, ovulation is followed by a luteal phase whether fertilization occurred or not. This luteal phase is characterized by elevated progesterone levels that are maintained for several weeks^36^. A luteal phase was defined as six or more consecutive fecal samples with a P4 value greater than 1.5 standard deviations above baseline mean (Supplementary Fig. 5). To assess if supraphysiological AMH affected the luteal phase of the cycle, we compared the mean number of luteal phases occurring every 6 months during the pre- and the post-treatment period for each group of cats. Luteal phase frequency was significantly reduced in the high-dose AAV9-fcMISv2 group (*P* = 0.0263), but not in the low-dose group or controls (Fig. 3g). Together, these results suggest that supraphysiological AMH suppresses ovulation and/or corpus luteum formation in the cat, resulting in a decreased frequency of its sequela, the luteal phase.

### Treatment with AAV9-fcMISv2 results in contraception in the cat

To assess the contraceptive potential of AAV9-fcMISv2 treatment, we initiated two 4-month-long mating trials at the 8- and 20-month post-treatment timepoints (Fig. 2a), using a different proven-breeder male for each trial. The male was group-housed with the nine females for 8 h/day, 5 days/week and continually video monitored to record all breeding interactions. Weekly transabdominal ultrasonography was performed to assess pregnancy status. For both trials, all control females conceived following their first breeding bout, defined as a period of consecutive days with at least one coital act per day (Table 1 and Supplementary Tables 1 and 2). In both trials, the controls gave birth to 2–4 healthy kittens in each litter. In contrast, no AAV9-fcMISv2-treated females gave birth during either trial and no gestational sacs or fetuses were observed at weekly ultrasound exams. Because no kittens were born from treated females, we did not assess maternal-fetal transmission of AMH. However, we established reference values for newborn AMH levels in a subset of male kittens (*n* = 3), with blood taken within 24 h of birth (from control mothers), which we measured at 0.254 ± 0.72 µg/ml.

**Table 1:** Breeding activity, ovulation, and pregnancy occurrence in AAV9-fcMISv2 treated cats versus controls during two separate mating trials

	Trial 1 (10/21/19–2/21/20)			Trial 2 (10/19/20–2/18/21)		
	Control	Low	High	Control	Low	High
	*n* = 3	*n* = 3	*n* = 3	*n* = 3	*n* = 3	*n* = 3
Number of females that allowed breeding	3	1	1	3	1	1
Total number of breeding bouts	3	6	1	3	3	1
Number of luteal phases that followed a breeding bout^a^	3	0	0	3	0	0
Number of pregnant females^a^	3	0	0	3	0	0
Total kittens produced^b^	10	0	0	11	0	0

Source data are provided as a Source Data file.

^a^Chi-square test of homogeneity: *P* = 0.0498 for both trials.

^b^Chi-square test of homogeneity: *P* < 0.0001 for both trials.

Two of the AAV9-fcMISv2-treated females did exhibit breeding activity during the trials (Table 1, Supplementary Tables 1 and 2). “Dolly” (low-dose group) allowed six breeding bouts during the first trial and three breeding bouts during the second. “Barbara” (high-dose group) allowed one breeding bout during each trial. No luteal phases were detected in fecal hormone analyses following any of the bouts (Table 1). No breeding behavior was observed from the other four treated females. Using a chi-square test of homogeneity, no differences were seen between groups in the number of females that allowed breeding or total breeding bouts. However, there were significant differences in the number of luteal phases that followed a breeding bout (*P* = 0.0498), as well as the number of females that became pregnant (*P* = 0.0498), and total kittens produced (*P* < 0.0001) for both mating trials (Table 1). These results strongly suggest that supraphysiological AMH inhibits induced ovulation, and results in contraception in the cat.

## Discussion

We have previously shown that IP injection of an AAV9 vector delivering human AMH in mice resulted in transduction of tissues such as skeletal muscles and liver, which in turn acted as in vivo bioreactors, secreting AMH for systemic delivery to the ovaries, causing ovarian suppression and contraception^8^. Similarly, herein, we used IM delivery of an AAV9 vector containing an optimized feline AMH (fcMISv2) transgene to evaluate its effects on reproductive cyclicity, mating, and fecundity in the female domestic cat. AAV9-fcMISv2 treatment resulted in the induction of supraphysiological levels of AMH protein in the serum, above the speculated contraceptive serum threshold of 0.25 µg/ml in mice^8^, for the 2 year duration of the study. While the AMH concentration in the serum declined over time, perhaps due to the turnover of shorter-lived transduced cells (e.g., liver cells), this decline eventually stabilized, suggesting continued expression by skeletal muscles that are efficiently transduced by AAV9^16^. Maintenance of long-term secretion of AMH by skeletal muscles was also supported by evidence of continued fcMISv1 expression in muscle tissue of “India” more than 4 years after treatment (Supplementary Fig. 2A).

Although the cat is classified as an induced ovulator (with copulation being the canonical inducer), spontaneous ovulation has been well documented in this species^37,38^. In AAV9-fcMISv2-treated cats, we observed a significant decrease in P4 levels and luteal phase frequency, which in turn strongly suggests a reduction in the high spontaneous ovulation rate often seen in group-housed cats^39^. Surprisingly, CNP levels increased while FST decreased during the post-treatment period only in the control group suggesting seasonal and/or male induced changes of these hormones produced by antral follicles^34,40–43^. It remains unclear if AMH could affect such seasonal or breeding-induced hormonal changes in the cat, representing a limitation of this study. Indeed, we did not observe any significant changes in inhibin A or B, follistatin, E2, or T levels following AAV9-fcMISv2 treatment in cats. Together, these hormonal data suggest that the mechanism of contraception of supraphysiological AMH in cats may be distinct from the one observed in mice, where follicles arrest at the pre-antral stage resulting in significant reductions in inhibin B, E2, and T, and a complete inhibition of estrous cycling^8^. Several studies have reported that co-treatment of AMH with FSH can inhibit the FSH-dependent induction of Cyp19a1 and E2 production in human granulosa-lutein cells^35,44–46^, and in ex vivo cultured ovaries from mice^47^. However, despite the purported role of AMH in inhibiting production of E2 and modulating pituitary gonadotropins directly^48,49^, our findings suggest that supraphysiological AMH does not suppress E2 levels in cats, potentially because pituitary feedback may restore its homeostasis even in the face of abnormal follicle development. Indeed, in mice, supraphysiological AMH induces contraception by suppressing both the activation of primordial follicles, and the maturation of pre-antral follicles by inhibiting granulosa cell differentiation and proliferation, causing a stall in primary and early secondary follicle development, and leading to a near complete absence of antral follicles^9^, yet E2 levels are only reduced by approximately half in that species^8^. In the cat, we hypothesize that supraphysiological AMH may also inhibit primordial follicle activation and preantral follicle maturation, but that some follicles escape AMH suppression to grow to an antral-like stage before stalling, where they are capable of producing normal levels of E2 under pituitary control.

While we favor the interpretation that the contraception observed herein is caused by a direct effect of AMH on the ovary, by inhibiting ovarian follicles to complete maturation and ovulate in response to an LH surge, we cannot rule out an impairment of the LH surge itself. Indeed, others have shown that AMH may also directly regulate gonadotropins in the pituitary, increasing the LH/FSH ratio^48–50^, suggesting AMH could also cause infertility with a mechanism analogous to the anovulation observed in women with polycystic ovarian syndrome (PCOS), who also experience elevated AMH and high LH/FSH ratio^51^. However, unlike PCOS in women^51^, AAV9-fcMISv2-treated cats in our study do not have elevated T. Alternatively, the elevated LH observed in treated cats could be a consequence of the reduced P4 secretion causing a mild hypergonadotropic hypogonadism phenotype^52^. Further studies will be necessary to examine the specific follicular populations present in AMH-treated cat ovaries and to evaluate if their antral follicles can respond to an induced LH surge, and if corpora lutea can form. Nonetheless, the hormonal and mating data collected herein strongly suggest that neither total follicular suppression nor complete inhibition of reproductive behavior may be necessary for contraception with AMH in the female domestic cat given that even animals with unchanged levels of inhibin B, inhibin A, FST, E2, and T (Fig. 3b, c and Supplementary Fig. 4A and B) that recorded estrous and mating events (Fig. 3d and Table 1) were still infertile.

In cats, high levels of AMH appeared to inhibit the occurrence of luteal phases following natural breeding as demonstrated in the two mating trials, suggesting impairment of induced ovulation. When estrous frequency was determined by assessing consecutive E2 elevations in fecal samples, no differences between the pre- and post-treatment periods were observed in AAV9-fcMISv2-treated cats (Fig. 3d). However, when estrus is defined behaviorally by the female permitting mounting and coitus, an effect of treatment can clearly be observed. All three control females mated repeatedly with both males, whereas four of the six treated females rebuffed every mating attempt by the breeder males during both mating trials (Table 1 and Supplementary Tables 1 and 2). Mate choice among females may have influenced breeding activity, but our use of two different breeder males (of greatly different phenotypes and ages) and the consistent receptiveness of randomly assigned control females to both males make this interpretation unlikely. We speculate that females which did not present estrous behavior following treatment with AAV9-fcMISv2 either had either insufficient peak E2 to elicit the behavior, or displayed increased sexual rejection as a result of the effect of AMH on progesterone-sensitive nitric oxide synthase expressing neurons in the hypothalamus, as reported in mice treated prenatally with AMH^53^. Further studies will be needed to examine the role of AMH on the hypothalamus and pituitary of the cat. Altogether, these data suggest that while supraphysiological AMH inhibits ovulation efficiently, its mechanism of ovarian suppression may allow sufficient estradiol production to elicit mating behavior in a subset of cats. A better understanding of the hormonal basis of estrous behavior in these cats may be necessary to mitigate potentially unwanted estrous behavior if an AMH-contraceptive is to be developed in companion animals. However, in feral animals, non-productive breeding may be beneficial for population control. Of greatest importance, however, is that despite the observed mating behavior, both AAV9-fcMISv2 breeding females failed to become pregnant, while all control females produced kittens in both mating studies.

Because AAV9-fcMISv2 vectored contraception is being proposed here as an alternative to ovariectomy and ovariohysterectomy in female cats, the long-term health of retained reproductive organs is of key consideration. Cystic endometrial hyperplasia-pyometra complex is a clinically relevant and potentially life-threatening disease in intact female cats. Ovarian hormones contribute to the pathogenesis, with ovulation and progesterone playing a primary role. The disease is characterized by hyperplasia of the endometrium, cystic dilation of endometrial glands, uterine inflammation, and purulent discharge (reviewed in^30^). A study assessing the histopathology of 106 reproductive tracts from clinically healthy cats presenting for elective ovariohysterectomy detected cystic endometrial hyperplasia in 21 (~20%) and pyometra in 2 (~2%) individuals^37^. The six treated females in this study received their AAV9-fcMISv2 injections ~4 years ago. Physical exams and transabdominal ultrasounds performed every 3 months and bloodwork assessed every 6 months have found no evidence of cystic endometrial hyperplasia-pyometra or aberrations in systemic blood parameters in any female. Because these females are still intact, we cannot yet evaluate their uterine histopathology. However, histological analysis of the reproductive tracts of the females from the first pilot study revealed that cystic endometrial hyperplasia did not develop in “Antilles”, who maintained high AMH levels throughout the study, but did develop in both females (“Catalina” and “India”) that produced anti-fcMISv1 antibodies and failed to maintain high AMH. Furthermore, the ovaries of “India”, the female that developed the strongest antibody response and had the lowest AMH expression, displayed multiple corpora lutea indicative of past spontaneous ovulations. These data suggest a possible protective effect of supraphysiological AMH in preventing cystic endometrial hyperplasia-pyometra in intact females by prevention of spontaneous ovulation.

In cats, our findings are most relevant for contraception, specifically by demonstrating that AAV9-fcMISv2 gene therapy can produce multi-year inhibition of ovulation, and pregnancy prevention with no apparent adverse health effects in treated females. These data also suggest that AMH can suppress luteal phases and ovulation without compromising E2 levels and its salutary effect on healthy longevity, which is an important consideration for long-term uses of AMH or AMHR2 agonists as contraceptives in women^54^. We propose that this single dose, non-surgical contraceptive could represent a viable alternative to surgical sterilization and provide veterinary practices, shelters, and animal control programs with a rapid and easily applied option to induce lifelong contraception of household and free-roaming female domestic cats.

## Methods

### Animals

Mice experiments were conducted with six weeks old outbred Nu/Nu (Crl:NU-Foxn1^nu^) nude mice (Gnotobiotic Mouse Cox7 Core), and were approved by the National Institute of Health and Harvard Medical School Institutional Animal Care and Use Committee, in accordance with the Massachusetts General Hospital approved experimental protocol 2014N000275. The mice were housed in 12 h light/12 h night conditions with room temperature and humidity maintained between 20–23 °C and 30–70%, respectively. Mice had unlimited access to Prolab® IsoPro® RMH 3000 (LabDiet, catalog # 5P76) rodent chow and water.

Domestic cats (*Felis silvestris catus*) were maintained in a research colony at the Cincinnati Zoo and Botanical Garden’s Center for Conservation and Research of Endangered Wildlife (CREW). All procedures were approved by the Institutional Animal Care and Use Committee (Identification Number 18–132) and the Cincinnati Children’s Hospital Medical Center Institutional Biosafety Committee (IBC 2018-0066). All cats in the main study (*n* = 9) and in the pilot study (*n* = 3) were sexually intact, nulliparous domestic short-haired females. They ranged from 6 to 12 months (main study) and from 6 to 7 years (pilot study) of age at the beginning of the study. Cats were group-housed under a 14:10 h light-dark cycle, fed Purina Pro Plan Adult Complete Essentials dry cat food (Nestle Purina Petcare), and provided access to fresh water *ad libitum*. Two proven breeder male cats (2 years old and 13 years old) used during the mating trials were singly housed in a separate room, but otherwise kept under the same conditions. Physical exams and blood work (complete blood count (CBC) and serum biochemistry panel) were performed prior to study enrollment.

### Transgene cloning and recombinant protein purification

We engineered the first-generation AAV9 vector (AAV9-fcMISv1) using the cat reference genome (V8.0 assembly) with sequence gaps filled with dog (*Canis lupus familiaris*) sequence, and optimized codon usage to reduce GC content and maximize expression in the cat using the GenSmart™ Codon Optimization algorithm (GenScript). We engineered a second-generation AAV9 vector (AAV9-fcMISv2) matching the updated cat reference genome (V9.0 assembly) also optimized with the GenSmart™ algorithm. Both fcMISv1 and fcMISv2 DNA and protein sequences are available in Supplementary Data 1, and sequence alignment was performed using the Clustal Omega Multiple Alignment Package^55^ (www.ebi.ac.uk/Tools/msa/clustalo/; accessed on December 2022).

Those same sequences were subcloned into a pcDNA3.1 expression vector with the introduction of a FLAG-tag following the endogenous cleavage site, so that the FLAG would be positioned at the N-terminus of the mature domain (termed AMH_c_) following enzymatic cleavage, for purification purposes as previously described^23,24^. Briefly, the pcDNA3.1 constructs were stably transfected into CHO-K1 cells (ATCC, catalog # CCL-61), and high expressing clones were derived. The selected clones for each construct were expanded into HyperFlask cell culture vessels (Corning), where they were maintained in DMEM containing 5% FBS, 1% penicillin-streptomycin and 1% L-glutamine, and Geneticin (G418, Thermofisher) at 1.1 g/L. The media conditioned for a week with confluent cells was used for purification. The FLAG-tagged AMH (FLAG-fcMISv1 and FLAG-fcMISv2) was purified by immunoaffinity chromatography using anti-FLAG M2 Affinity gel (Sigma-Aldrich). The beads were rinsed with 5 column volumes of 1X Tris Buffered Saline (TBS) pH 7.4 (Boston Bioproducts) and eluted with 3X FLAG peptide (Sigma-Aldrich). Elution fractions were pooled, concentrated in a 15 ml spin column (Millipore), and dialyzed in a 3.5 K MWCO Slide-A-Lyzer cassettes (Thermofisher Scientific) in 1X PBS at 4 °C overnight. The protein was cleaved in vitro using plasmin (Sigma-Aldrich) with a plasmin to AMH ratio of 1:18 (weight:weight) and incubated 4 h at 37 °C, followed by heat inactivation of plasmin at 57 °C for 7 min.

### AAV9-fcMIS treatment

Mice received a single AAV9-fcMISv2 IP injection at 5e12 or 1e13 vg/kg, or 5e12 vp/kg of AAV9-empty vector. Blood was collected from mice by cheek vein puncture prior to AAV9 injection and weekly afterwards. Mice were euthanized via CO_2_ asphyxiation followed by cervical dislocation 1 month after injection. Their ovaries were harvested and fixed in formalin overnight before being mounted in paraffin blocks for sectioning.

Female cats were randomly assigned to one of three treatment groups: control (5e12 vp/kg AAV9-empty vector, *n* = 3), low-dose (5e12 vg/kg AAV9-fcMISv2, *n* = 3), or high-dose (1e13 vg/kg AAV9-fcMISv2, *n* = 3), using the random number generator function in Microsoft Excel. On Day 0 of the study (11-Feb-2019), cats were anesthetized using a ketamine/dexmedetomidine combination with partial reversal using atipamezole. A single injection (0.51-1.30 ml, total volume) was administered in the right caudal thigh muscles of each cat. In the first pilot study, the three female cats received 5e12 vg/kg of AAV9-fcMISv1. Cats were housed singly for 7 days under Biosafety Level 2 (BSL-2) containment before returning to group-housing.

### Health monitoring

Cats were assessed daily for general well-being. Physical exams and blood work (CBC and serum biochemistry panel) were performed two weeks prior to treatment, at Day 0 (before AAV9-fcMISv2 treatment), and repeated every 3 months through Year 1 of the study, and every 6 months thereafter. Injection sites were examined daily for 14 days, weekly for 2 weeks, and monthly thereafter.

### Mating trials

Two 4-month-long mating trials (which began 8- and 20-months post-treatment) were initiated using a different proven-breeder male for each trial. The male was group-housed with all females for eight hours per day, 5 days per week (Monday–Friday), and continually video monitored for breeding interactions. Weekly transabdominal ultrasound exams were performed on females to assess pregnancy status. Interactions were assessed by video review and scored as a successful breeding (defined as confirmed intromission and appropriate behavioral responses from the female) or breeding attempt (defined as male attempting to mount female or successful mount without intromission). The identity of each female for an interaction was determined by a member of the animal keeper staff. If females conceived following breeding, they were removed from the mating trial, transferred into individual maternity caging at ~Day 50 of pregnancy and closely monitored through expected time of parturition.

### Cat sample collection

Viral shedding. Fecal and urine samples were collected daily from individual cats during BLS-2 housing. Oral swabs were performed at Days 0 (pre-treatment), 2, 7, and 14. Whole blood (jugular vein, cephalic vein, or lateral saphenous vein) was collected in microtainer EDTA tubes at Days 0 (pre-treatment), 2, 7, 14, 21, 28, and monthly thereafter through Month 6. Urine, oral swabs, and blood samples were transferred into 1.8 ml cryovials. Fecal samples were sealed in plastic bags. All samples were stored at −20 °C until analysis.

Serum samples. Venous blood samples were collected from adult female cats at Days 0 (pre-treatment), 2, 7, 14, 21, 28, and monthly thereafter through Year 2, and from any newborn kittens born to study females within 24 h of birth. Blood was collected into serum separator tubes, allowed to clot for approximately fifteen minutes, and centrifuged. The recovered serum was transferred into 1.8 ml cryovials and stored at −80 °C until analysis.

Fecal samples. Fecal samples were collected three non-consecutive days per week beginning 6 months prior to AAV9 treatment and concluding 2 years post-treatment. To facilitate identification of fecal samples from group-housed females, a unique combination of commercially available food-grade dye (Wilton Industries) and/or glitter (Dixon Ticonderoga Company) was fed to each cat in a small amount of Purina Pro Plan Adult Complete Essentials canned cat food (Nestle Purina Petcare) each day preceding a sample collection day. Samples were sealed in plastic bags, labeled with name and date collected, and stored at −20 °C until processing.

Reproductive tracts. Female cats enrolled in the pilot study underwent surgical ovariohysterectomy 42 months post-injection. The removed reproductive tracts were fixed in formalin, processed in an automated tissue processor (Leica TP1020) and embedded in paraffin blocks. Five-microns sections were stained with Mayer’s hematoxylin (Dako) and eosin (Sigma Aldrich), coverslipped with Cytoseal 60 (Thermo Fisher Scientific), and examined under a light microscope for histological analysis.

### Enzyme immunoassays

Fecal estrogens and progestogens. Fecal samples were lyophilized in their plastic bags using a freeze dryer (Labconco Corp), pulverized into a fine powder, and then weighed (250 ± 5 mg) into labeled 15 ml polypropylene conical tubes. Each of the samples was then extracted by adding 2.5 ml of 90% ethanol (or a 1:10 w:v) overnight on a mechanical rocker (≥12 h). Extracted samples were then centrifuged at 1000 *g* for 15 min, supernatants were pipetted off, and samples stored in 2.0 ml cryovials at −20 °C until analysis.

Procedures for all enzyme immunoassays (EIAs) were modified from previously published methods^56^. A polyclonal antibody produced against 17β-estradiol was used in conjunction with a horseradish peroxidase (HRP) ligand to determine estrogens (E2), whereas the Arbor Assays progesterone mini-kit (Arbor Assays) was used to quantify progestogens (P4). For both assays, samples and standards were analyzed in duplicate.

Serum luteinizing hormone. Diluted serum samples (1:5) were analyzed utilizing a double antibody EIA adapted from Graham et al. ^57^ NIH-bovine LH standards, controls, and samples were added to plate wells in duplicate. After overnight incubation, biotinylated NIH-ovine LH was added to all wells and allowed to compete for 4 h at room temperature. After competition, plates were then incubated with streptavidin-peroxidase. The EIA was validated for cat serum by demonstrating parallelism between dilutions of pooled serum and the standard curve. Furthermore, bovine LH (used for standards) was added to cat serum samples and a dose-response curve was generated. Intra- and inter-assay coefficients of variation were 3.5% and 8%.

### In situ RNA hybridization

In situ RNA hybridization was performed using RNAscope 2.5 HD Reagent Kit (RED, ACD Bio)^58^. Skeletal muscle and liver tissue sections from “India” were hybridized with probes designed to specifically identify the fcMISv1 transgene, following the manufacturer’s instructions. Briefly, following xylene deparaffinization and heat-induced epitope retrieval, the tissues were hybridized for 2 h and processed for standard signal amplification steps, and chromogen development. Slides were finally counterstained with hematoxylin, air-dried and cover slipped with EcoMount.

### ELISAs

C-reactive protein (CRP). CRP levels were measured in serum samples from cats injected with AAV9-fcMISv1 using the Cat CRP ELISA kit (Abcam). Samples were diluted 1:8,000 and assayed following the manufacturer’s protocols.

Direct AMH neutralizing antibody ELISA. We designed and optimized a new direct capture protein ELISA assay to measure anti-fcMISv1/v2 IgG in cat serum. Briefly, in this ELISA, recombinant FLAG-tagged fcMISv1 or v2 (5 μg/ml capture protein) purified from conditioned media from CHO-K1 cells is directly coated onto the ELISA plate (Immulon HB2 ELISA plate, Thermofisher Scientific). Standard wells were coated with whole molecule cat IgG (Rockland Antibodies and Assays), in eight 3-fold dilutions in coating buffer. Control and blank wells received just coating buffer. The plate was then incubated 30 min at RT and overnight at 4 °C. Two rinses were performed, and the plate was blocked for 2.5 h with 200 µl/well 1% Bovine Serum Albumin (Jackson Laboratories) + 7.5% normal goat serum (Abcam) in phosphate buffered saline tween-20 (PBST). Samples were diluted by a factor of 100 in blocking buffer, added to the plate, and incubated for 1 h at RT. After 5 washes, the plate was incubated for 1 h in the dark at 4 °C with goat anti-feline IgG (H + L) HRP (Novus Biologicals, catalog # NBP1-73347) 1:10,000 in PBST. Plate was rinsed five times and the enzyme substrate reaction performed.

AMH. AMH levels were measured using the AMH Gen II ELISA (Beckman Coulter). Briefly, cat sera were diluted in sample diluent as follows: all control, Day 0 low, and Day 0 high-dose cats, 1:10; subsequent low-dose cats, 1:500 to 1:1000 (except Dolly, 1:100); high-dose cats, 1:1000 to 1:2000. Samples from newborn male kittens were diluted 1:100. Manufacturer’s instructions were followed for the remainder of the ELISA.

Reproductive hormone blood measurements. C-type natriuretic peptide (CNP) levels were measured in cat serum samples diluted 1:4 using the Mouse CNP/NPPC ELISA kit (Novus Biologicals). Inhibin A, inhibin B, follistatin and testosterone were measured in undiluted serum samples using the Cat Inhibin B, Cat Inhibin beta A chain, Canine Follistatin (FST) and General Testosterone ELISA kits, respectively (all from MyBioSource). Samples were assayed following the manufacturer’s protocols.

### Follicle counts

Formalin-fixed paraffin-embedded mice ovaries were serially cut at 5 microns, and one section out of every five was kept for follicle quantification^8^. Following H&E staining, slides were individually photographed and follicles with a visible oocyte nucleus were quantified by two independent observers. Briefly, follicles with one layer of squamous granulosa cells were qualified as primordial, one layer of cuboidal granulosa cells as primary, several layers of cuboidal granulosa cells as secondary, and ones presenting an antrum as antral. A correction factor of five was applied to obtain the final number of follicles. One ovary from 4 to 5 mice per group was quantified.

### Protein extraction and western blot

Freshly dissected tissues or transfected cells were homogenized in RIPA Lysis Buffer System with protease inhibitor cocktail and PMSF (Santa Cruz Biotechnology), sonicated twice for 15 s at 11% amplitude and centrifuged 20,800 × *g* for 15 min at 4 °C. Supernatant was collected and protein content measured using Pierce BCA Protein Assay (ThermoFisher Scientific). Samples were prepared with either 50 or 100 μg protein extract, 4X sample buffer and RIPA buffer and electrophoresis in Nupage 4–12% Bis Tris 1.5 mm gels (ThermoFisher Scientific). Proteins were transferred to a Nu-PAGE PVDF membrane and blocked with 5% milk for one hour. Overnight incubation in primary, goat anti-AMH C-terminus antibody, AMH C-20 (Santa Cruz, catalog # sc-6886) 1:200 in 5% milk was followed by a 1.5-2 h incubation in donkey, anti-goat IgG HRP (Jackson ImmunoResearch Laboratories, catalog # 705-035-003) 1:2000.

### Urogenital ridge assay

A urogenital ridge Müllerian duct regression assay was performed to measure the activity of recombinant AMH proteins^23,25^. Briefly, E14.5 female rat embryos urogenital ridges were dissected and set in culture on agar coated steel grids at the media/air interface and treated with conditioned media (fcMISv1, fcMISv2, LR-hsMIS at 5 μg/ml) for 72 h in humidified 5% CO_2_ at 37 °C. For fcMISv1, the protein was purified using FLAG immunoaffinity chromatography followed by plasmin cleavage. For fcMISv2, conditioned culture medium was collected after 72 h, concentrated, and adjusted to 5 µg/ml based on ELISA measurements, and used to treat the urogenital ridges. After incubation, the samples were fixed in Zamboni buffer, and paraffin embedded. Ridge sections (8 μm) were stained with H&E. Scores from 0 (no regression) to 5 (complete regression of the Müllerian duct) were then attributed by two independent individuals.

### Statistics and reproducibility

Sample size, randomization, and blinding. Based on previous published results by our group, the differences in the number of primary, secondary, and antral follicles following AAV9-MIS treatment were expected to be large^8^. We performed power calculations using data from this study and other pilot experiments for which female mice were treated with AAV9-MIS. Assuming an alpha of 5% (a confidence interval of 95%) and a conservative beta of 50%, we estimated that *n* = 4 mice per group would be sufficient to reach statistical significance in follicle counts. The AAV9-fcMISv2 contraceptive was predicted to result in complete infertility in treated cats thus *n* = 3 females per group involved in two mating trials was deemed powerful enough to measure difference in reproductive output. Female mice and cats were randomly assigned to treatment groups. Investigators were not blinded to the treatment group allocation of the cats and for assessment of most outcomes. Scoring of male-female interactions during mating trials was performed by blinded investigators.

Follicle counts, hormonal and viral titer data. Analysis and graphing were performed using Prism 9.0 software (GraphPad). Average follicle counts were analyzed by one-way ANOVA followed by a Dunnett post-hoc test and compared to the AAV9-empty group for each follicular stage. LH, CNP, inhibin A, inhibin B, follistatin, and testosterone data were first log-transformed to fit a normal distribution. Data from the transition and post-treatment periods were then compared using an unpaired two-tailed Student’s t test for each group of cats. Estradiol and progesterone data were transformed and compared following the same procedure, but pre- and post-treatment periods were compared. No data were excluded from the analyses. Significant differences were declared when *P* < 0.05.

Fecal hormone metabolites. Hormone baseline values were calculated for each female based on the 6-month pre-treatment sampling period. Data were analyzed using the *R* statistical package *hormLong* (version1.0)^59^. For each individual, baseline estrogens (E2) and progestogens (P4) values were calculated using an iterative process excluding all points greater than the mean plus 1.5 standard deviations^57,60^. An estrous phase was defined as two or more consecutive fecal samples with an E2 value greater than 1.5 standard deviations over baseline. A luteal phase was defined as six or more consecutive fecal samples with a P4 value greater than 1.5 standard deviations over baseline^56^. Number of estrous phases, number of luteal phases, and mean fecal metabolite concentrations were compared between two time periods: 6 months pre-treatment versus 2–24 months post-treatment, after a 2-month post-treatment transition phase. Periods of pregnancy and lactation were excluded from analyses. To account for the different lengths of time for each sampling period, estrous phases are reported as the number of estrous phases in a 1-month period (total number of estrous phases/number of days in sampling period x 30 days) and luteal phases are reported as the number of luteal phases in a 6-month period (total number of luteal phases/number of days in sampling period x 180 days). Data were analyzed as a randomized complete block design, using an ANOVA, where Period and Treatment Group (and their interaction) are fixed effects, and the individual animals are included as a random effect (blocks). The Tukey’s multiple mean comparison test was used for pairwise comparisons. Analyses were performed using SAS® Studio software (Release: 3.8, Enterprise Edition, SAS Institute Inc., Cary, NC, USA). Significant differences were declared when *P* < 0.05.

Mating trials. Number of breeding females, total breeding bouts, number of luteal phases post-breeding, number of pregnant females, and total kittens produced were compared among treatments within each mating trial using a Chi-square test of homogeneity, where the null hypothesis was a uniform distribution across treatments. Analyses were performed using SAS® Studio software. No data were excluded from the analyses. Significant differences were declared when *P* < 0.05.

### Reporting summary

Further information on research design is available in the Nature Portfolio Reporting Summary linked to this article.

## Supplementary information

Supplementary Information

Description of Additional Supplementary Files

Supplementary Data 1

Reporting Summary

## Data Availability

The main data supporting the findings of this study are available within the article and its Supplementary Information, Supplementary Data and Source Data files. Additional details on datasets or protocols that support the findings of this study will be made available by the corresponding authors upon request. A reporting summary for this article is available as Supplementary Information file. The GenBank accession codes for the cat reference genome assemblies referenced in this work are GCA_000181335.2 (Felis_catus_8.0) and GCA_000181335.3 (Felis_catus_9.0), and they are accessible at https://www.ncbi.nlm.nih.gov/assembly/GCF_000181335.2/ and https://www.ncbi.nlm.nih.gov/assembly/GCF_000181335.3/ respectively. Source data are provided with this paper.

## Source data

Source Data
