# Supplementary material for: Durable contraception in the female domestic cat using viral-vectored delivery of a feline anti-Müllerian hormone transgene
Source: Nat Commun. 2023 Jun 6;14:3140. doi: 10.1038/s41467-023-38721-0 (PMC10244415; doi:10.1038/s41467-023-38721-0)
Supplement: Supplementary file 1 — Supplementary Information [file 41467_2023_38721_MOESM1_ESM.pdf]

Supplementary Information

Durable contraception in the female domestic cat using viral-vectored delivery of a feline anti-Müllerian hormone transgene

Lindsey M. Vansandt[1], Marie-Charlotte Meinsohn[2], Philippe Godin[2], Nicholas Nagykery[2], Natalie Sicher[2], Motohiro Kano[2], Aki Kashiwagi[2], Maeva Chauvin[2], Hatice D. Saatcioglu[2], Julie L. Barnes[1], Amy G. Miller[1], Amy K. Thompson[1], Helen L. Bateman[1], Elizabeth M. Donelan[1], Raquel González[1], Jackie Newsom[1], Guangping Gao[3], Patricia K. Donahoe[2], Dan Wang[3], William F. Swanson[1] & David Pépin[2]**

[1]Center for Conservation and Research of Endangered Wildlife (CREW), Cincinnati Zoo & Botanical Garden; Cincinnati, OH, USA.

[2]Pediatric Surgical Research Laboratories, Massachusetts General Hospital, Department of Surgery, Harvard Medical School; Boston, MA, USA.

[3]Horae Gene Therapy Center, University of Massachusetts Medical School; Worcester, MA, USA.

*Correspondence and requests for materials should be addressed to DP (dpepin@mgh.harvard.edu) or WFS (bill.swanson@cincinnatizoo.org).

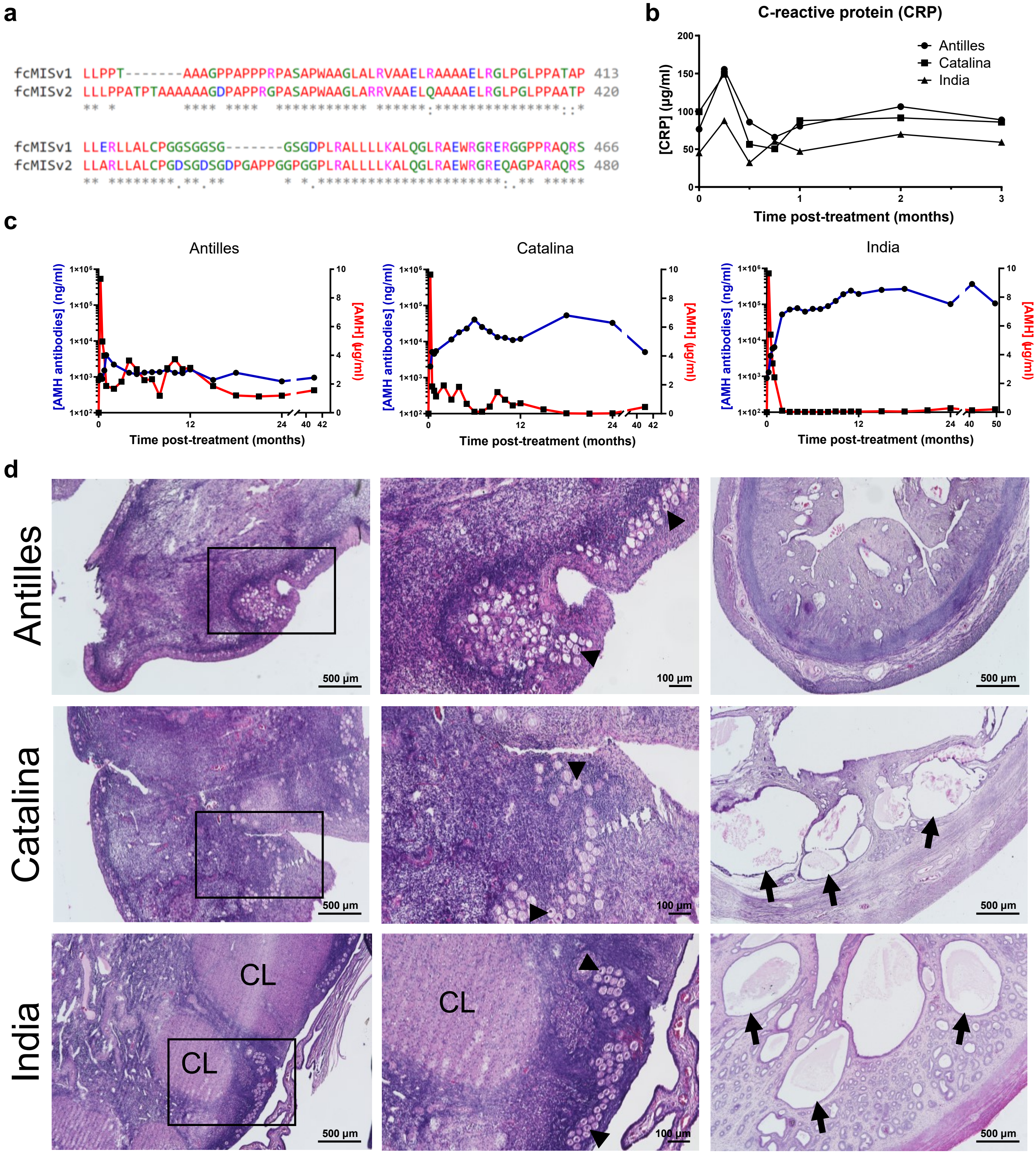

Supplementary Fig. 1 Pilot study with a first-generation feline transgene (fcMISv1) in female cats. **a** Sequence alignment of the divergent section of the feline AMH protein from the domestic cat genome release versions 8.0 (fcMISv1) and 9.0 (fcMISv2) using the ClustalOmega Multiple Alignment Package. Matching amino acids are denoted with an asterisk (*). **b** C-reactive protein (CRP) levels in the serum of three cats following treatment with 5e12 vg/kg fcMISv1. **c** Serum anti-fcMISv1 antibody titers (blue line, circles) and AMH (red line, squares) in the same female cats throughout the study. **d** H&E staining of ovary and uterus showing corpora lutea (CL), primordial follicles (arrowheads), and endometrial cysts (arrows). Female cats underwent ovariohysterectomy 42 months after treatment with 5e12 vg/kg fcMISv1. Source data are provided as a Source Data file.

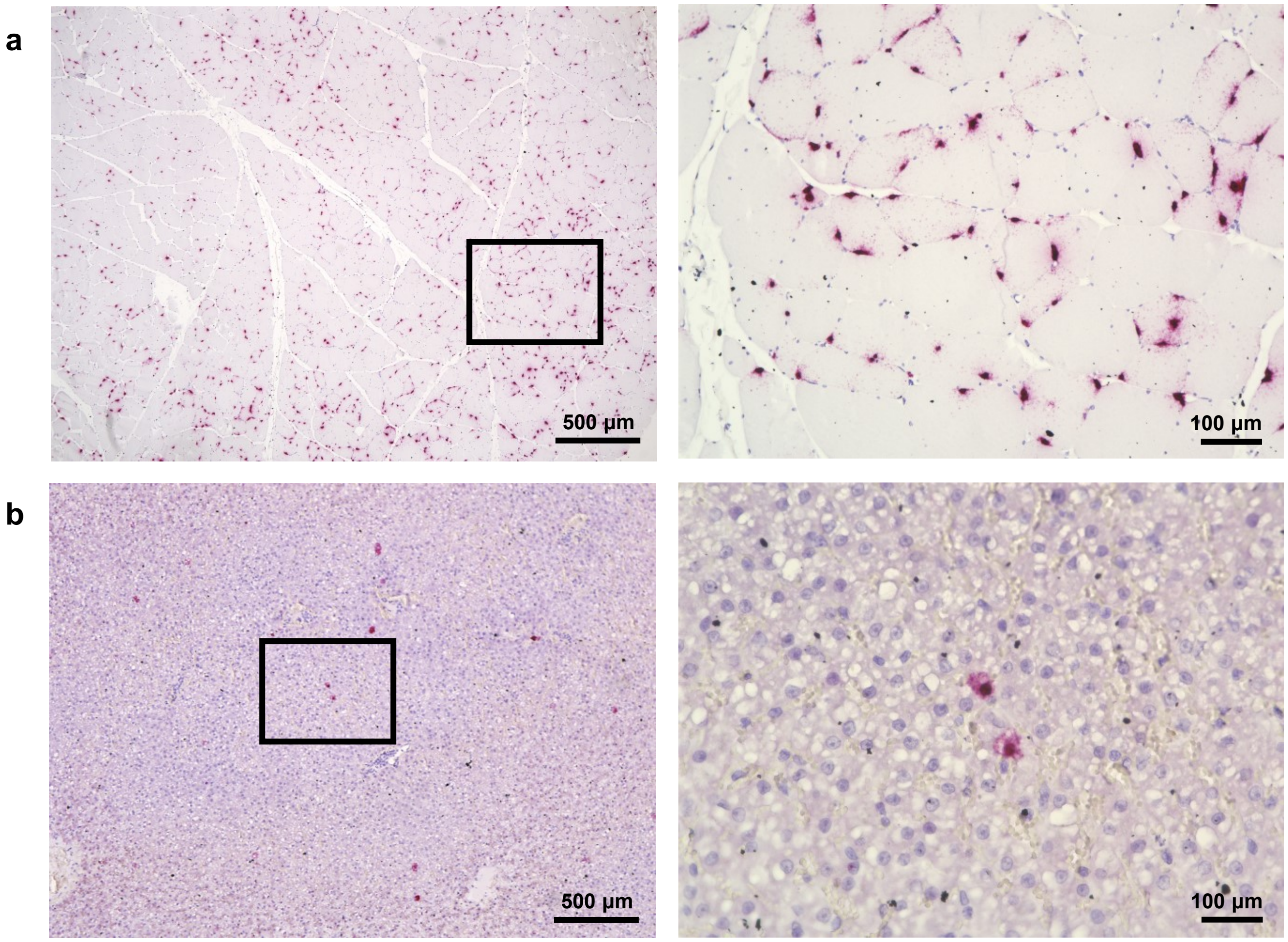

Supplementary Fig. 2 AMH transgene expression in post-mortem tissues of "India" 49.5 months after injection with 5e12 vg/kg of AAV9-fcMISv1. *In situ* RNA hybridization of skeletal muscle (**a**) and liver (**b**) tissues showing AMH positive cells (stained in red). Counterstaining with hematoxylin.

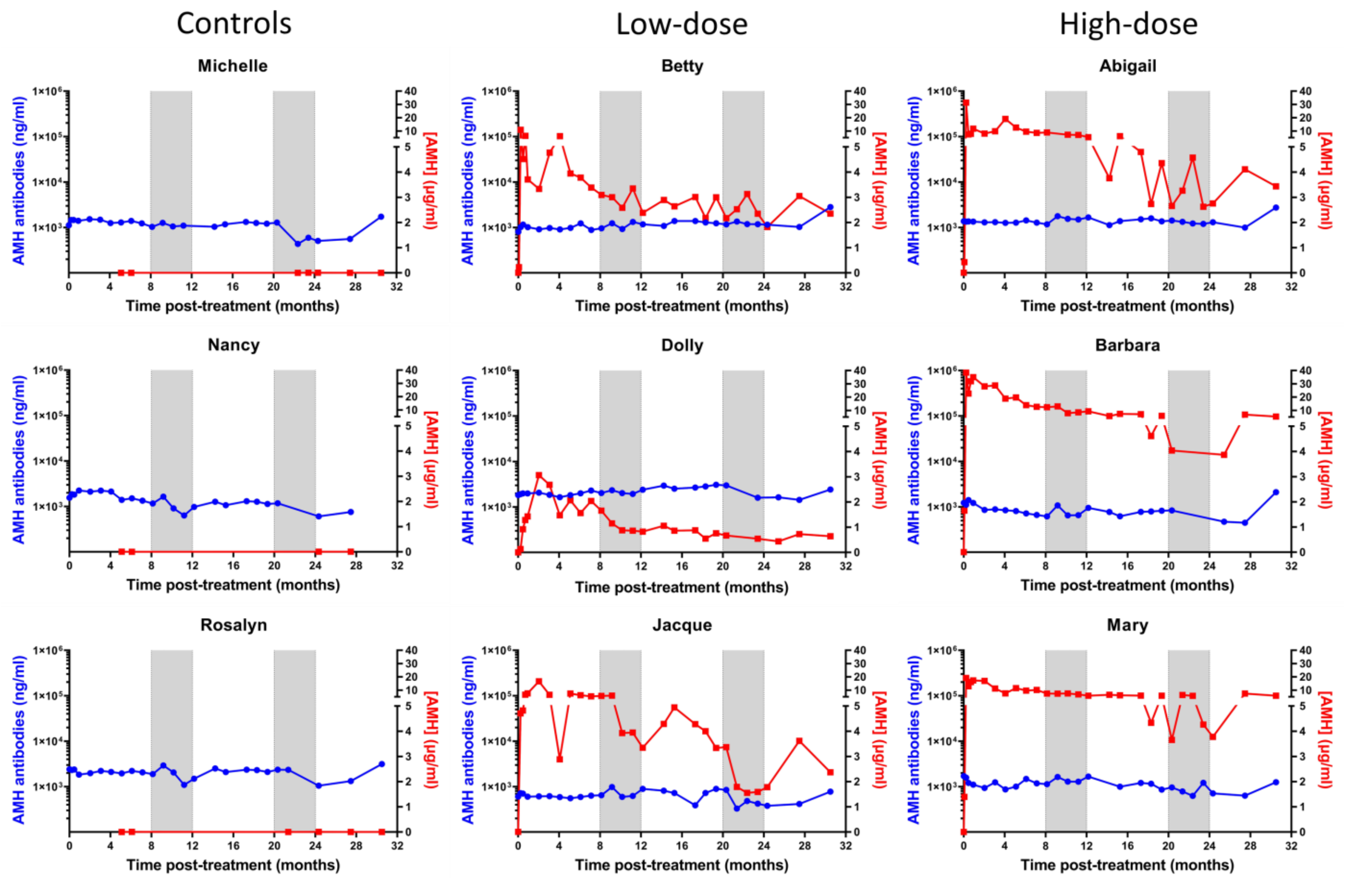

Supplementary Fig. 3 Individual serum anti-fcMISv2 antibody titers (blue line, circles) and AMH (red line, squares) profiles in female domestic cats during the AAV9-fcMISv2 contraceptive study. Controls (left column) were injected with 5e12 viral particles per kilogram (vp/kg) of AAV9-empty vector. Low-dose (middle column) and high-dose (right column) females were treated with 5e12 and 1e13 viral genomes per kilogram (vg/kg) of AAV9-fcMISv2, respectively. Mating trial periods are shaded in grey. Source data are provided as a Source Data file.

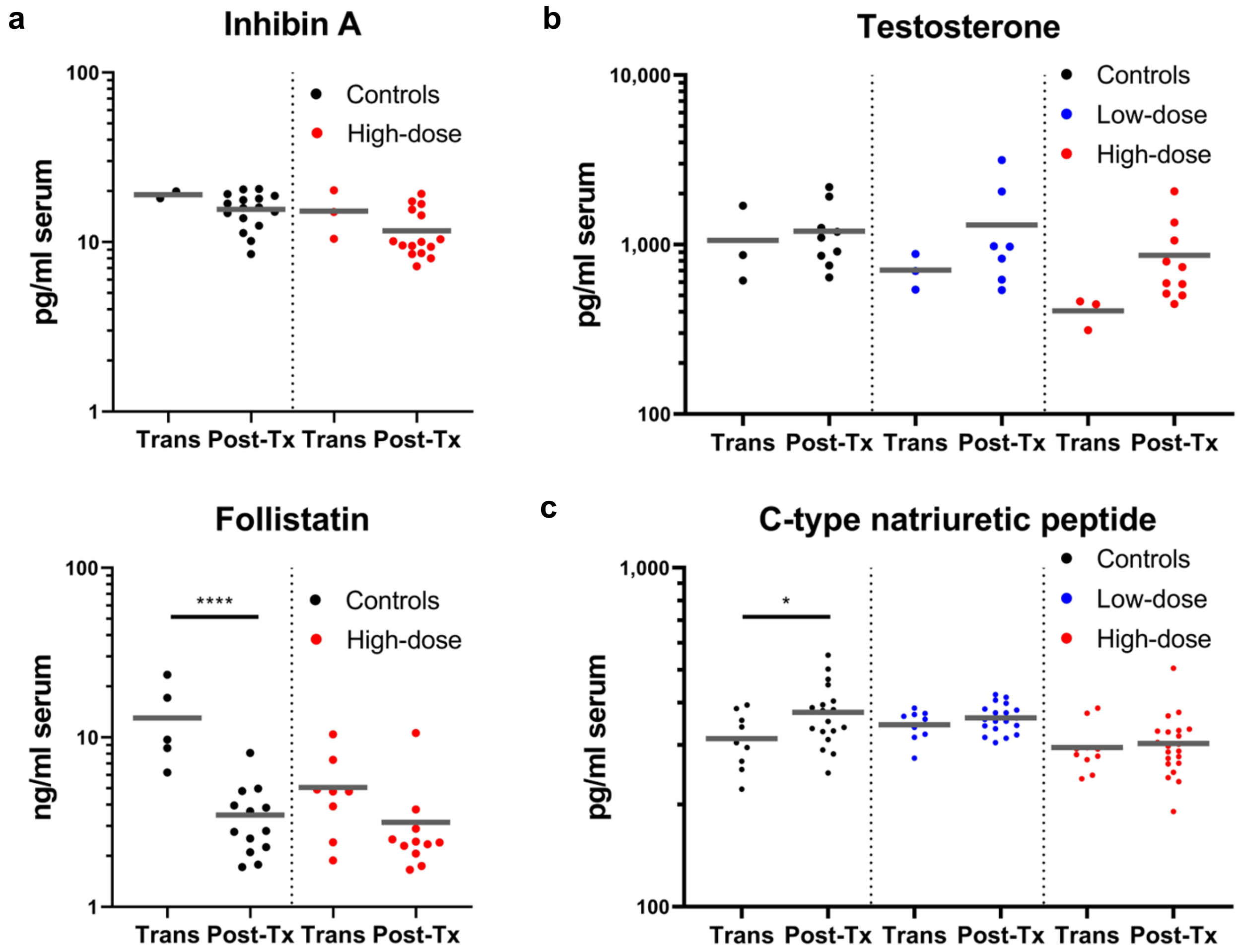

Supplementary Fig. 4 Evaluation of several markers of ovarian activity in the serum of female cats. Sexually mature female domestic cats were injected intramuscularly with 5e12 viral particles per kilogram (vp/kg) of AAV9-empty vector (controls, black), or with either 5e12 (low-dose, blue) or 1e13 (high-dose, red) viral genomes per kilogram (vg/kg) of AAV9-fcMISv2. Serum inhibin A and follistatin (**a**), testosterone (**b**) and c-type natriuretic peptide (**c**) levels were assessed by ELISA and compared between the transition period (Trans, 0-2 months post-injection) and the post-treatment period (Post-Tx, 2-24 months post-injection) for each group of cats. Horizontal lines indicate the mean. n=3 biologically independent animals for each experiment. Log-transformed data were compared using an unpaired two-tailed Student's t test for each group of cats: *P < 0.05; ****P < 0.0001. P < 0.0001 for follistatin. P = 0.0453 for c-type natriuretic peptide. Source data are provided as a Source Data file.

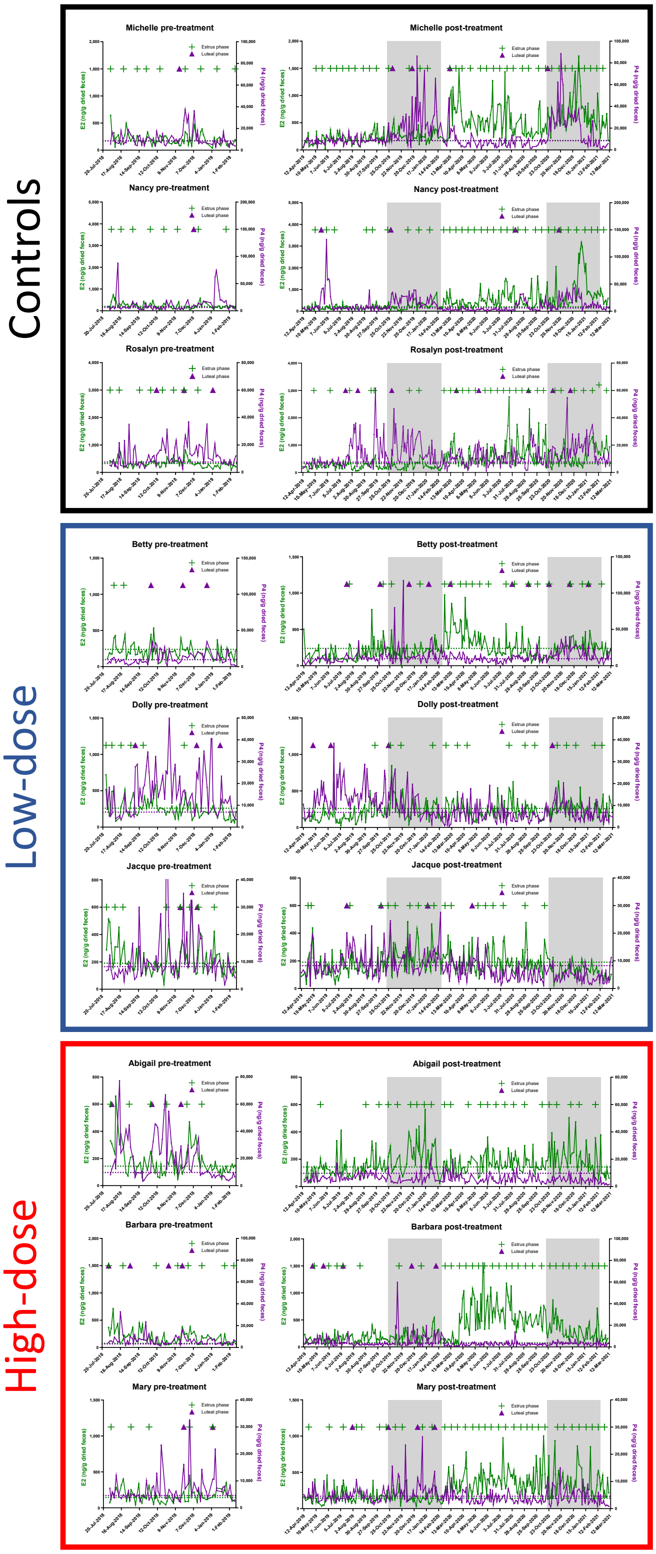

Supplementary Fig. 5 Fecal sex steroids and assessment of cyclicity in individual cats injected with 5e12 vp/kg of empty AAV9 vector (black frame), or treated with either 5e12 (low-dose, blue frame) or 1e13 (high-dose, red frame) vg/kg of AAV9-fcMISv2. Concentrations of estradiol (green line) and progesterone (purple line) were assessed in dried fecal samples collected through the pre-(left column) and post-(right column) treatment periods. Consecutive peak steroid concentrations (>1.5 times over the baseline mean) were used to estimate estrus phases (when E2 peaks in ≥2 consecutive samples, green plus sign) and luteal phases (when P4 peaks in ≥6 consecutive samples, purple triangle). Mating trial periods are shaded in grey. Source data are provided as a Source Data file.

Supplementary Table 1. Breeding activity, estrus, and luteal phase occurrence, and pregnancy in AAV9-treated cats during the first mating trial (10/21/19 – 2/21/20).

Treatment	Cat	Dates (M/D/YY)	Description of breeding	S	A	Estrous phase	Luteal phase	Comments
Control	Michelle	12/11/19 – 12/13/19	Multiple successes and few attempts	12	2	Yes	Yes	Resulted in pregnancy
	Nancy	10/22/19 – 10/25/19	Multiple successes and few attempts	20	5	Yes	Yes	Resulted in pregnancy
	Rosalyn	10/28/19 – 10/31/19	Multiple successes and few attempts	18	4	No	Yes	Resulted in pregnancy
Low	Betty			0	0			No breeding behavior observed
	Dolly	10/30/19 – 11/1/19	Multiple successes and attempts	8	6	Yes	No	Luteal phase was ongoing from 10/22/19
		11/21/19 – 11/22/19	Two successes	2	0	Yes	No	Luteal phase was ongoing from 10/22/19
		12/16/19 – 12/17/19	Multiple successes and attempts	4	4	No	No	Intermittent elevated P4 seen
		1/2/20 – 1/10/20	Multiple successes and attempts	10	13	No	No	Intermittent elevated P4 seen
		1/23/20 – 1/31/20	Mostly attempts with some success at end	4	17	Yes	No	Estrus observed at end of breeding bout
		2/7/20	Mostly successes	6	1	Yes	No	Same estrus phase as last bout
			**Total**	**34**	**41**			
	Jacque	11/6/19	No attempt at breeding but male displayed interest in female	0	0	Yes	No	
High	Abigail			0	0			No breeding behavior observed
	Barbara	11/28/19	Single attempt	0	1	No	No	
		1/28/20 – 1/31/20	Multiple successes and attempts	12	5	No	No	Sporadic high P4 before and after breeding
			**Total**	**12**	**6**			
	Mary	1/2/20	Multiple attempts, no successes	0	4	No	No	

S: Successful breeding, A: Attempted breeding
Source data are provided as a Source Data file.

Supplementary Table 2. Breeding activity, estrus, and luteal phase occurrence, and pregnancy in AAV9-treated cats during the second mating trial (10/19/20 – 2/18/21).

Treatment	Cat	Dates (M/D/YY)	Description of breeding	S	A	Estrous phase	Luteal phase	Comments
Control	Michelle	10/22/20 – 10/27/20	Multiple successes	11	0	Yes	Yes	Resulted in pregnancy
	Nancy	11/6/20 – 11/11/20	Multiple successes	44	0	Yes	Yes	Resulted in pregnancy
	Rosalyn	11/23/20 – 11/26/20*	Multiple successes and few attempts	19	2	Yes	Yes	Resulted in pregnancy
Low	Betty			0	0			No breeding behavior observed
	Dolly	11/5/20 – 11/9/20	Multiple successes and attempts	4	5	Yes	No	Luteal phase was ongoing from 10/27/20
		11/26/20 – 12/2/20	Multiple successes and attempts	11	8	Yes	No	Luteal phase was ongoing from 10/27/20
		12/7/21 – 1/15/2021	Mostly attempts	1	4	No	No	
			**Total**	**16**	**17**			
	Jacque			0	0			No breeding behavior observed
High	Abigail			0	0			No breeding behavior observed
	Barbara	10/22/20 – 11/24/20	Multiple successes and attempts	125	26	Yes (3)	No	Sporadic high P4 before and after breeding
	Mary			0	0			No breeding behavior observed

S: Successful breeding, A: Attempted breeding
*Successful breeding also seen during pregnancy on 1/4/21 (1), 1/13/21 (1) and 1/14/21 (2), with an estrus peak detected on 1/9/21.
Source data are provided as a Source Data file.
