# Supplementary material for: Durable contraception in the female domestic cat using viral-vectored delivery of a feline anti-Müllerian hormone transgene
Source: Nat Commun. 2023 Jun 6;14:3140. doi: 10.1038/s41467-023-38721-0 (PMC10244415; doi:10.1038/s41467-023-38721-0)
Supplement: Supplementary file 2 — Description of Additional Supplementary Files [file 41467_2023_38721_MOESM2_ESM.pdf]

Description of Additional Supplementary Files

File Name: Supplementary Data 1
Description: fcMISv1 and fcMISv2 DNA and protein sequences
