# Supplementary material for: Durable contraception in the female domestic cat using viral-vectored delivery of a feline anti-Müllerian hormone transgene
Source: Nat Commun. 2023 Jun 6;14:3140. doi: 10.1038/s41467-023-38721-0 (PMC10244415; doi:10.1038/s41467-023-38721-0)
Supplement: Supplementary file 4 — Reporting Summary [file 41467_2023_38721_MOESM4_ESM.pdf]

nature portfolio

Corresponding author(s): David Pepin

Last updated by author(s): Apr 7, 2023

Reporting Summary

Nature Portfolio wishes to improve the reproducibility of the work that we publish. This form provides structure for consistency and transparency in reporting. For further information on Nature Portfolio policies, see our Editorial Policies and the Editorial Policy Checklist.

Statistics

For all statistical analyses, confirm that the following items are present in the figure legend, table legend, main text, or Methods section.

n/a	Confirmed	
☐	☒	The exact sample size (*n*) for each experimental group/condition, given as a discrete number and unit of measurement
☐	☒	A statement on whether measurements were taken from distinct samples or whether the same sample was measured repeatedly
☐	☒	The statistical test(s) used AND whether they are one- or two-sided<br>*Only common tests should be described solely by name; describe more complex techniques in the Methods section.*
☒	☐	A description of all covariates tested
☒	☐	A description of any assumptions or corrections, such as tests of normality and adjustment for multiple comparisons
☐	☒	A full description of the statistical parameters including central tendency (e.g. means) or other basic estimates (e.g. regression coefficient) AND variation (e.g. standard deviation) or associated estimates of uncertainty (e.g. confidence intervals)
☐	☒	For null hypothesis testing, the test statistic (e.g. *F*, *t*, *r*) with confidence intervals, effect sizes, degrees of freedom and *P* value noted<br>*Give P values as exact values whenever suitable.*
☒	☐	For Bayesian analysis, information on the choice of priors and Markov chain Monte Carlo settings
☒	☐	For hierarchical and complex designs, identification of the appropriate level for tests and full reporting of outcomes
☒	☐	Estimates of effect sizes (e.g. Cohen's *d*, Pearson's *r*), indicating how they were calculated

Our web collection on statistics for biologists contains articles on many of the points above.

Software and code

Policy information about availability of computer code

Data collection	No software was used for data collection
Data analysis	P4 and E2 fecal metabolite analysis was performed using the R statistical package hormLong (version 1.0). Estrous and luteal phase frequency analysis was performed using the SAS Studio 3.8 software. All other data analyses and graphing were performed using Prism 9.0 software (GraphPad).

For manuscripts utilizing custom algorithms or software that are central to the research but not yet described in published literature, software must be made available to editors and reviewers. We strongly encourage code deposition in a community repository (e.g. GitHub). See the Nature Portfolio guidelines for submitting code & software for further information.

Data

Policy information about availability of data

All manuscripts must include a data availability statement. This statement should provide the following information, where applicable:

- Accession codes, unique identifiers, or web links for publicly available datasets
- A description of any restrictions on data availability
- For clinical datasets or third party data, please ensure that the statement adheres to our policy

The main data supporting the findings of this study are available within the article and its Supplementary Information, Supplementary Data and Source Data files. The GenBank accession codes for the cat reference genome assemblies referenced in this work are GCA_000181335.2 (Felis_catus_8.0) and GCA_000181335.3 (Felis_catus_9.0), and they are accessible at https://www.ncbi.nlm.nih.gov/assembly/GCF_000181335.2/https://www.ncbi.nlm.nih.gov/assembly/

GCF_000181335.2/ and https://www.ncbi.nlm.nih.gov/assembly/GCF_000181335.3/ respectively.

Human research participants

Policy information about studies involving human research participants and Sex and Gender in Research.

Reporting on sex and gender	N/A
Population characteristics	N/A
Recruitment	N/A
Ethics oversight	N/A

Note that full information on the approval of the study protocol must also be provided in the manuscript.

Field-specific reporting

Please select the one below that is the best fit for your research. If you are not sure, read the appropriate sections before making your selection.

[x] Life sciences [ ] Behavioural & social sciences [ ] Ecological, evolutionary & environmental sciences

For a reference copy of the document with all sections, see nature.com/documents/nr-reporting-summary-flat.pdf

Life sciences study design

All studies must disclose on these points even when the disclosure is negative.

Sample size	Based on previous published results by our group (DOI: 10.1073/pnas.1620729114), the differences in the number of primary, secondary and antral follicles following AAV9-MIS treatment were expected to be large. We performed power calculations using data from this study and other pilot experiments for which female mice were treated with AAV9-MIS. Assuming an alpha of 5% (a confidence interval of 95%) and a conservative beta of 50%, we estimated that a sample size of 4 should be sufficient to reach statistical significance. In comparison, most ovarian biology studies assessing follicle counts in mice uses n=3-6 per group (e.g., DOIs: 10.1038/s41467-022-31759-6, and 10.1186/s13048-020-00724-6).<br><br>The main comparison was the reproductive output of control AAV9-empty animals versus treatment with AAV9-fcMISv2. We did not have useful preliminary data in the cat to base our calculations given the first pilot experiment (AAV9-fcMISv1) used an immunogenic transgene, but our hypothesis based on serum levels of AMH predicted complete infertility after AAV9-fcMISv2 treatment as observed in mice. Thus, we expected the treated females to have no litters, precluding power calculations for differences in litter sizes, and elected for groups of n=3 as a conservative group size that minimize animal research involvement.
Data exclusions	As stated in the Methods section, periods of pregnancy and lactation were excluded from fecal hormone metabolites analyses. No other data were excluded from the analyses.
Replication	Follicle counts of mice ovaries were performed by two independent investigators to ensure reproducibility. Female cats were treated with two dose groups, and two mating trials were performed to ensure reproducibility. All attempts at replication were successful.
Randomization	Female mice and cats were randomly assigned to experimental groups using the random number generator function in Microsoft Excel.
Blinding	- Investigators were not blinded to the treatment group allocation of mice for follicle counts. The greatly reduced ovarian size of mice treated with AAV9-fcMISv2 preclude blinding since group allocation is easily perceived during necropsy and later during histological examination of the ovaries.<br>- Investigators were not blinded to the treatment group allocation of the cats. The three authors who administered the treatment to the cats were also the veterinarians in charge of data collection. However, male-female interactions during mating trials were scored by blinded investigators.

Reporting for specific materials, systems and methods

We require information from authors about some types of materials, experimental systems and methods used in many studies. Here, indicate whether each material, system or method listed is relevant to your study. If you are not sure if a list item applies to your research, read the appropriate section before selecting a response.

Materials & experimental systems

n/a	Involved in the study
	☒ Antibodies
	☒ Eukaryotic cell lines
☒	Palaeontology and archaeology
	☒ Animals and other organisms
☒	Clinical data
☒	Dual use research of concern

Methods

n/a	Involved in the study
☒	ChIP-seq
☒	Flow cytometry
☒	MRI-based neuroimaging

Antibodies

Antibodies used	- AMH C-20 (Santa Cruz, catalog # sc-6886).<br>- Goat anti-feline IgG (H+L) HRP (Novus Biologicals, catalog # NBP1-73347).<br>- Donkey anti-goat IgG HRP (Jackson ImmunoResearch Laboratories, catalog # 705-035-003).
Validation	AMH C-20 was validated in mice tissues using recombinant AMH produced in our laboratory as a positive control.

Eukaryotic cell lines

Policy information about cell lines and Sex and Gender in Research

Cell line source(s)	CHO-K1 from ATCC
Authentication	Authentication was performed at ATCC prior to shipping.
Mycoplasma contamination	All cell lines tested negative to mycoplasma.
Commonly misidentified lines (See ICLAC register)	No commonly misidentified cell lines were used in the study

Animals and other research organisms

Policy information about studies involving animals; ARRIVE guidelines recommended for reporting animal research, and Sex and Gender in Research

Laboratory animals	- Mouse experiments were conducted with six weeks old female Nu/Nu nude mice (Gnotobiotic Mouse Cox7 Core).They were housed in 12 hours light/12 hours night conditions with room temperature and humidity maintained between 20-23 °C and 30-70%, respectively. Mice had unlimited access to Prolab® IsoPro® RMH 3000 (LabDiet, catalog # 5P76) rodent chow and water.<br><br>- Cat experiments were performed using domestic cats (Felis silvestris catus) maintained in a research colony at the Cincinnati Zoo and Botanical Garden’s Center for Conservation and Research of Endangered Wildlife (CREW). Cats from the pilot study (AAV9-fcMISv1) were 5.6-7.0 years old at treatment. Cats from the main study (AAV9-fcMISv2) were 1.2-1.3 years old at treatment. Cats were fed a daily diet of Purina Pro Plan Adult Complete Essentials Chicken & Rice Formula Dry Cat Food (Nestle Purina Petcare, UPC 38100-13154). They also received small amounts of Purina Pro Plan Adult Complete Essentials Beef & Carrots Entree Canned Cat Food (Nestle Purina Petcare, UPC 38100-16888) each day preceding a feces sample collection.
Wild animals	No wild animals were used in this study
Reporting on sex	Findings apply only to females as the aim of the study was to test a female contraceptive.
Field-collected samples	No field collected samples were used in this study
Ethics oversight	Experiments in mice were approved by the National Institute of Health and Harvard Medical School Institutional Animal Care and Use Committee, in accordance with the Massachusetts General Hospital approved experimental protocol 2014N000275. Experiments in cats were approved by the Institutional Animal Care and Use Committee (Identification Number 18-132) and the Cincinnati Children's Hospital Medical Center Institutional Biosafety Committee (IBC 2018-0066).

Note that full information on the approval of the study protocol must also be provided in the manuscript.
